# microRNA Regulation in Estrogen Receptor-Positive Breast Cancer and Endocrine Therapy

**DOI:** 10.1186/s12575-018-0082-9

**Published:** 2018-09-11

**Authors:** Erin W. Howard, Xiaohe Yang

**Affiliations:** Julius L. Chambers Biomedical/Biotechnology Research Institute, Department of Biological and Biomedical Sciences, North Carolina Central University, North Carolina Research Campus, 500 Laureate Way, NRI 4301, Kannapolis, North Carolina 28081 USA

**Keywords:** Estrogen receptor alpha (ERα), ER^+^ breast cancer, microRNA (miRNA), miRNA biogenesis, Endocrine therapy resistance, ER-receptor tyrosine kinase (RTK) crosstalk

## Abstract

As de novo and acquired resistance to standard first line endocrine therapies is a growing clinical challenge for estrogen receptor-positive (ER^+^) breast cancer patients, understanding the mechanisms of resistance is critical to develop novel therapeutic strategies to prevent therapeutic resistance and improve patient outcomes. The widespread post-transcriptional regulatory role that microRNAs (miRNAs) can have on various oncogenic pathways has been well-documented. In particular, several miRNAs are reported to suppress ERα expression via direct binding with the 3’ UTR of *ESR1* mRNA, which can confer resistance to estrogen/ERα-targeted therapies. In turn, estrogen/ERα activation can modulate miRNA expression, which may contribute to ER^+^ breast carcinogenesis. Given the reported oncogenic and tumor suppressor functions of miRNAs in ER^+^ breast cancer, the targeted regulation of specific miRNAs is emerging as a promising strategy to treat ER^+^ breast cancer and significantly improve patient responsiveness to endocrine therapies. In this review, we highlight the major miRNA-ER regulatory mechanisms in context with ER^+^ breast carcinogenesis, as well as the critical miRNAs that contribute to endocrine therapy resistance or sensitivity. Collectively, this comprehensive review of the current literature sheds light on the clinical applications and challenges associated with miRNA regulatory mechanisms and novel miRNA targets that may have translational value as potential therapeutics for the treatment of ER^+^ breast cancer.

## Background

Estrogen receptor-positive (ER^+^) breast cancer accounts for nearly 65% of breast cancers. In particular, due to the association of hormone levels and ER activation/regulation, the ER^+^ breast cancer subtype is particularly prevalent in postmenopausal women taking hormone replacement therapy. The activation of ERα, a major transcription factor, promotes the transcription of a number of target genes that regulate oncogenic processes, including cell cycle progression, cell survival, and epithelial/luminal cell differentiation. Although anti-estrogens, selective ER modulators (SERMs), and selective ER downregulators (SERDs), such as tamoxifen and fulvestrant, are leading treatment strategies that can block mitogenic estrogen activity and have significantly improved ER^+^ breast cancer patient outcomes, therapeutic resistance remains a significant clinical challenge. Therefore, understanding the mechanisms that regulate estrogen/ER-mediated oncogenic activity will shed light on novel therapeutic targets to more effectively treat ER^+^ breast cancer.

Within the past 15 years, substantial research has highlighted the role of microRNA (miRNA) regulation that can contribute to ER^+^ breast cancer risk or prevention. In particular, studies have not only demonstrated that miRNAs are targets of ERα/hormonal signaling, but have also shown that ERα is a regulatory target of multiple miRNAs. In this review, we highlight miRNA signatures that are associated with ER^+^ and ER^−^ breast cancer subtypes, as well as key tumor suppressor miRNAs and oncomiRs that are deregulated in ER^+^ breast cancer and modulate therapeutic resistance. Importantly, we also discuss the potential clinical applications of novel therapeutic strategies targeting miRNAs, and their role in the treatment of therapeutic-resistant ER^+^ breast cancers.

## miRNAs Associated with ER^+^ Versus ER^−^ Breast Cancer Subtypes

The identification miRNA profiles that are associated with specific cancers or predict clinical outcomes has been a major research focus in the past decade or more. In particular, miRNA expression profiling of human breast cancer subtypes, as classified by ER, progesterone receptor, and ErbB2/Her2 receptor statuses, has revealed miRNA signatures that not only correlate with the molecular subtype, but can also serve as potential prognostic markers and indicators of patient responses to facilitate personalized treatment approaches. As summarized in Table 1, numerous reports have identified miRNAs that are differentially expressed in ER^+^ versus ER^−^ breast cancer samples. Many of the miRNAs that are downregulated in ER^+^ breast cancer are tumor suppressor miRNAs, such as miR-206 [1, 2], that function to suppress cell proliferation and survival. The inhibition of tumor suppressor miRNAs consequently contributes to the oncogenic phenotypes associated with ER^+^ breast cancer, such as a high proliferative index in the intrinsic Luminal B subtype of ER^+^ breast cancers [3]. Indeed, the development of targeted therapeutics to upregulate these particular tumor suppressor miRNAs may serve as a promising strategy to treat ER^+^ breast cancer. In contrast, several oncomiRs have consistently been reported to be upregulated in ER^+^ breast cancer, such as miR-10b and miR-21 [4–6]. In particular, the tumor suppressor *PTEN*, which dephosphorylates PIP_3_ and in turn blocks PI3K/Akt signaling, is a direct target of many oncomiRs that are overexpressed in ER^+^ breast cancer (Table 1). Thus, the attenuation of *PTEN*-mediated blockage of the PI3K/Akt signaling by the aberrant overexpression of oncomiRs in ER^+^ breast cancer contributes to downstream Akt signaling, which promotes oncogenic cell growth, survival, and migration. The targeted inhibition of these oncomiRs could also provide alternative treatment options for ER^+^ breast cancer patients. As discussed in the following sections, miRNAs play an integral role in the regulation of estrogen/ERα signaling and many miRNAs can also be modulated by ERα activation. Yet, miRNA-mediated regulation of ERα expression/activity and other oncogenic signaling pathways are linked to resistance to standard first-line endocrine therapies.

**Table 1:** Differentially expressed miRNAs in ER^+^ vs. ER^−^ breast cancers and their major direct mRNA targets

miRNAs	Expression levels in ER^+^ (vs. ER^−^) breast cancer	Direct miRNA targets [117]
let-7a-c/f	High [4]	*CDK6, KRAS, NRAS, HRAS, ITGB3,* ***NF2*** *, HMGA1/2, EWSR1,* *DICER* *,* *AGO1* *,* *LIN28A/B* *, CASP3/8/9, PARP1, IL6/10, E2F2,* ***CCND1*** */2, CDC34, CDC25A, EZH2, WNT1, MAPK4K4, IRS2,* ***IGF1R*** *, TGFBR1, AKT2,* ***MYC*** *, NUMB*
miR-9	Low [118]	*ITGB1, RCOR1, FOXO1/3, CDH1, MAPK1/3, MMP9, TAZ, NOTCH2, HES1, CBX7, E2F1, RAB34, NFKB1, SIRT1, CCNG1, SOCS5, CREB1,* *DICER1* *,* ***NF1*** *, CXCR4, TGFBR2, BECN1*
miR-10a/b	High [4, 5]	*MAP3K7,* ***EPHA4*** *, HOXA1, HOXB3, ACTG1,* ***PTEN*** *, USF2, PIK3CA/G,* ***SERPINE1*** *, MMP14,* ***NCOR2*** *, CKDN1A/2A, KLF4, PPARA,* ***NF1*** *, PLK1, CCNA2, ZEB1*
miR-18a/b	High [57] Low [2, 4, 11]	***ESR1*** *, KRAS,* ***PTEN*** *, CTGF, NR3C1, HIF1A, TGFBR2, SMAD2/4, HSF2, ATM,* *DICER* *,* ***BCL2*** *, IRF2, RUNX1, MEF2D, CBX7, TNFAIP3, FOXN1*
miR-19a/b	Low [83]	***ESR1*** *, HOXA5,* ***PTEN*** *,* ***CCND1*** *, ERBB4, ATXN1, KAT2B, SOCS1, TGFBR2, BMPR2, KIT, TLR2, TNF, TNFAIP3, FOXP1, BTG1, PIK3CA, MAP3K5, CUL5, AKT1, FGFR2, HIPK1*
miR-21	High [6]	*RASGRP1, CDC25A,* ***BCL2*** *, JAF1, SMARCA4, SPRY2, DUSP10, TIMP3, SOX2/5, MTAP, DOCK5/7, RECK, TGFBR2/3,* ***PTEN*** *, E2F1, TGFBI,* ***SP1*** *, APAF1, BTG2,* ***PDCD4*** *, RHOB, BMPR2,* ***NCOA3*** *, TP63, MSH2/6, TIAM1, EGFR, ERBB2, ICAM1, PPARA, NTF3,* ***COL4A1*** *, SMAD7, MAP2K3, MAT2A/2B, STAT3, LRP6, FZD6, BMI1, SOCS1, FOXO1, CASP8, VEGFA*
miR-22	Low [7]	*PTMS, ERBB3, ARPC5,* ***BMP7*** *, PPARA,* ***ESR1*** *,* ***NCOA1*** *, HDAC4/6, HMGB1,* ***SP1*** *, RAB5B, TET2,* ***CDKN1A*** *, WNT1, SIRT1, NET1, TIAM1, MMP14, SNAI1, CXCR2, AKT1, CDK6*
miR-25	Low [4]	*BCL2L11, CCL26, KLF4, CDKN1C, KAT2B,* ***TP53*** *, CDH1,* ***MDM2*** *,* ***PTEN*** *, EZH2, SMAD7, ERBB2*
miR-26a/b	High [36]	*HMGA1/2, CCND2, CCNE1/2,* ***ESR1*** *, CDK6, CDC6,* ***PTEN*** *, EZH2, SERBP1, SMAD1/4, REB1, MAP3K2, GSK3B, NOS2, CHD1, FGF9, ATM, HGF, IGF1,* *LIN28B* *, DNMT3B, WEE1, ADAM17, CHEK1, ITGA5, DUSP4/5, NRAS, E2F2, PTGS2, JAG1,* ***MYC*** *, GATA4, TAB1, RB1, COX2*
miR-29b	High [36]	*TGFB1/3, HDAC4, COL1A1, COL3A1,* ***COL4A1/2*** *, COL5A2,* ***SP1*** *, CDK6, PPP1R13B,* ***PTEN*** *, DNMT1, DNMT3A/B, MCL1,* ***BCL2*** *, VEGFA, TET1/2, CDC42, MMP2/9/15/24, ADAM12, BMP1, HMGA2, GSK3B, INFG, PIK3R1, CCND2, SNAI3, AKT2/3, ITGA6,* ***GATA3*** *, PDGFRA, PDGRA-C, STAT3,*
miR-30a-d	High [4, 36]	*DTL, SMAD1, CDK6, NOTCH1, BECN1, SNAI1, PIK3CD, PRDM1, ABL1,* ***VIM*** *,* ***ESR2*** *, RUNX2, BCL9, SOX4, CBX3,* ***TP53*** *, NCAM1, DNMT1, ITGB3, ATF1, CCND2, CCNE1/2, RPA1, TET1, NOTCH1, KLF9, CAT, ATG12,* ***SERPINE1*** *, DLL4, PDGFRB,* ***BCL2*** *, EIF5A2, ADAM12, HOXA1, SOCS1/3, RUNX2, CASP3, PAK1, MCL1, FOXO3, EZH2*
miR-93	Low [4, 119]	***CDKN1A*** *, TP53INP1, E2F1, VEGFA, ITGB8, KAT2B, TUSC2,* ***PTEN*** *, LATS2, TGFBR2, DAB2, SMAD7, ZBTB4, CXCL8,* ***PDCD4*** *, ANG, PTENP1, NEDD4L, MMP3, ZNRF3,* ***FOXA1***
miR-106b	Low [4]	*ITCH, APP,* ***CDKN1A*** *, E2F1, KAT2B, RB1,* ***PTEN*** *, APC, CASP7,* ***JAK1*** *, SETD2, SMAD7, STAT3, ZBTB4, TWIST1, HIF1A, TRIM8, RUNX3, DAB2, PTENP1, CYBB, TNFSF10A/11*
miR-130a	High [4]	*HOXA5/10, ATXN1, KLF4, PPARG,* ***ESR1*** *,* *DICER1* *, RUNX3, RAB5A, SMAD4, TNF, TGFBR2, TGFB1, PPARA,* ***MYC*** *,* ***PTEN*** *, DLL4, MAP3K12, MECP2, MET*
miR-135b	Low [120]	*APC, KLF4, CASR, PPP2R5C, LATS2, SMAD5, MID1, MTCH2, BMPR2, TGFBR1, FOXO1*
miR-142	Low [4]	*RAC1, ARNTL, TGFBR1/2, BOD1, PROM1, ROCK2, CCNT2, TAB2, HMGA1/B1, PTPN23, TP53INP1, IL1A, HSPA1B, SMAD3, ABCG2, LGR5, ZEB1, SIRT1, HIF1A, SOCS1,* ***PTEN*** *, DOCK6*
miR-145	High [121]	*BNIP3, KLF4/5, SOX2/9, MUC1,* ***CDKN1A*** *, ITGB8, STAT1, YES1, CLINT1, IRS1/2, VEGFA, HOXA9,* ***MYC*** *, FLI1, IFNB1,* ***IGF1R*** *, FZD7, CDK4/6,* ***SERPINE1*** *,* ***ESR1*** *, JAG1, NEDD9, PAK4, ERG, NRAS, ADAM17, CDH2, EPAS1, ETS1, CD44, BRAF, SMAD2/3, TGFBR3, CTNND1,* ***SP1/7*** *, TNFSF13, DDX6, ARF6, ADD3, HMGA2, ROCK1, HDAC11, SENP1, NAIP, TUG1, TGFB2, EGFR, ACTB*
miR-148	Low [4]	*DNMT1/3B, TGIF2, MCL1, IRS1,* ***BCL2*** *, ITGA5/B8, ROCK1, PIK3CA, NRAS, CSF1, CDC25B, MAP3K4/9, MMP7, WNT1/10B, CDKN1B,* ***SERPINE1*** *, SMAD2, MET, USP4, STAT3, ALCAM, TGFB2, AKT2, BAX, CYBB, NRP1,*
miR-150	Low [4]	*MYB, MUC4, EGR2, ZEB1, EP300,* ***TP53*** *, CBL,* ***SP1*** *, CREB1, STAT1/5B, CTNNB1, NANOG, MMP14, ZNF350,* ***IGF1R*** *, BIRC5*
miR-155	Low [4]	*TAB2, SOCS1/6, MSH2/6, MLH1, DET1, SMAD1/2/5, ZNF652, ZIC3, BACH1, APC, TRIM32, RHOA, TP53INP1, SPI1, FOXO3, RUNX2,* ***JUN*** *, ETS1, ICAM1, IRAK3, MYB, SKI, SOX6, FADD, BCL6, NOS3,* ***CCND1*** *, NFKB1, E2F2,* ***MYC*** *, DOCK1, RAD51,* ***PTEN*** *, ERBB2, RPTOR, TFAM, STAT1, SIRT1,* ***TP53***
miR-185	Low [36]	*RHOA, CDC42, SIX1, DNMT1, EPAS1, ARC, NFATC3, VEGFA, MZB1, HMGA1/2,* ***IGF1R*** *, DUSP4, CASP14, EPHB2, SMAD7, TRIM29, TGFB1, AKT1, CCNE1, CDK6, ATR, EZH2*
miR-187	Low [4]	*TNF, BCL6, CRMP1,* ***CYP1B1*** *, FOXA2, ALDH1A3*
miR-190	Low [120]	*IGF1,* ***PTEN*** *,* ***BCL2*** *, KCNQ5, MARK2, FOXP2*
miR-191	High [36]	*MDM4, TMC7, NDST1, SOX4, CDK6/9, SATB1, CEBPB, BASP1, NOTCH2, EGR1, CCND2*
miR-193b	High [2]	***ESR1*** *,* ***CCND1*** *, PLAU, PRAP1, MCL1, ETS1, MAX, KRAS, RAD51, MYB,* ***NF1*** *, SMAD3, STMN1*
miR-199a/b	High [4]	*MET, HIF1A, SMARCA2, CD44, EZH2, IKBKB, MAPK1, JUNB, DDR1, MAP3K11, CAV1/2, ERBB2, ERBB3, MTOR, SIRT1, PTGS2, HSPA5, ATF6, ERN1, CDH1/2, ZHX1, HGF, BECN1, IGF1, NFKB1, VEGFA, SNAI1, GSK3B, WNT2, FZD4/6, JAG1, HK2, TFAM, CCR7, TGFB2, PIK3CD, PAK4, CDK7, ITGA3, YAP1, MAP4K3m TGFBR1, FLT1, KDR, FOXA2, SLC27A1, AKT1, MAPK8/9/14, HES1, SET, DDR1, PAK4,*
miR-206	Low [1, 2, 36]	*MET,* ***NOTCH3*** *,* ***ESR1*** *, PAX3, CCND2, CDK4, NR1H3, KRAS, SFRP1,* ***CCND1*** *, ANXA2, NR4A2, SMAD2, TWF1, CCL2, VEGFA, SOD1, AKT1*
miR-212	Low [36]	*RB1, MECP2, TJP1, PEA15, PTCH1, KCNJ2, RBP2,* ***MYC*** *, ACHE, PXN, SOX4, SGK3, SMAD2, MAF, CCNA2/B1,* *AGO1*
miR-214	High [4]	*EZH2, XBP1,* ***PTEN*** *, MAP2K3, MAPK1/8, ING4, TWIST1, GALNT7,* ***TP53*** *, CTNNB1, JAG1, FGFR1, NRAS, BIRC5, CDK3/6, RAB15, E2F2, ITCH, SUFU, CPD, PIM1*
miR-217	High [120]	*SIRT1, ROBO1,* ***PTEN*** *, EZH2, E2F3, DACH1, FOXO3, PTPN14, DMNT1,* ***IGF1R***
miR-218	High [120]	*LAMB3, RICTOR, BIRC5/6, LASP1, IKBKB,* ***SP1*** *, VOPP1, ACTN1, ROBO1, SFRP2, HOXB3, DKK2, TOB1, CDK6, BMI1, HMGB1, LEF1, SLIT3, PDGFRA, GLI2, RUNX2, CDH2,* ***TFF1*** *, EGFR, LGR4, SMO, E2F2, CDC27, KIT, BCL9*
miR-221	High [2]	*CDKN1B/C, BMF, FOXO3,* *DICER1* *, KIT, TMED7, ETS1, BBC3, DKK2,* ***DDIT4*** *, TIMP3, ICAM1, FOS,* ***ESR1*** *, TICAM1,* ***PTEN*** *, TRPS1, WEE1, ZEB2, RB1, APAF1, RECK, SIRT1,* ***MDM2*** *, MGMT, SOCS1/3, ARF4,* ***CXCL12*** *, IL6R, BECN1, RUNX1/2, DVL2, PIK3R1, MMP2, RAD51*
miR-224	High [4]	*KLK10, CXCR4, CDC42, SMAD4, DIO1,* ***NCOA6*** *, FOSB, API5, CDH1, CASP3/7, ATG5, MTOR, FUT4, KRAS,* ***BCL2*** *, PAK2*
miR-299	Low [120]	***CDKN1A*** *,* ***SPP1*** *, IGF1*
miR-342	High [4, 120, 122, 123]	*GEMIN4,* ***BMP7*** *, DNMT1, ID4, SREBF1/2, CTBP2, BIRC6, E2F1*
miR-375	High [118]	*TIMM8A, PDK1, JAK2, YAP1, MTDH, RASD1,* ***SP1*** *, MAP3K8, LDHB, CIP2A,* ***TP53*** *, ERBB2,* ***IGF1R*** *, PIK3CA, DEPTOR, RUNX3, MALAT1, MYCN*
miR-519a	Low [124]	***CDKN1A*** *,* ***PTEN*** *, YES1,* *DICER1* *, TIMP1, RB1, FOXF2, STAT3*
miR-520g	Low [122]	*VEGFA, SMAD7, MMP2*

mRNAs in bold are related to ER regulation and ER target genes. Underlined mRNAs are involved in miRNA biogenesis regulation

## miRNAs and Estrogen/ERα Regulation

### miRNAs that Regulate ERα

Many miRNAs that are associated with ER^+^/ER^−^ breast cancer subtypes also directly target or indirectly regulate *ESR1*/ERα. To this end, many *ESR1*-targeting miRNAs are downregulated in ER^+^ breast cancer or are upregulated in endocrine therapy-resistant breast cancers (Table 2). In this section, the role of these *ESR1*-targeting miRNAs in ER signaling regulation will be discussed in context with ER^+^ breast carcinogenesis and prevention.

**Table 2:** miRNAs that regulate *ESR1*/ERα

miRNA	Effects on *ESR1*/ERα and known mechanisms of ER regulation	References
miR-1	Suppresses *ESR1* expression	[7]
miR-9	Suppresses *ESR1* expression ➢ Directly targets *ESR1*	[7, 11, 125]
miR-18a/b	Suppresses *ESR1* expression ➢ Directly targets *ESR1* Inhibits ERα transcriptional activity	[7, 11, 57, 82]
miR-19a/b	Suppresses *ESR1* expression ➢ Directly targets *ESR1* Inhibits ERα transcriptional activity	[57]
miR-20a/b	Suppresses *ESR1* expression ➢ Directly targets *ESR1* ➢ Directly targets *NCOA3* Inhibits ERα transcriptional activity	[57]
miR-22	Suppresses *ESR1* expression ➢ Directly targets *ESR1* ➢ Directly targets *SP1*	[7–9, 11]
miR-26a/b	Suppresses *ESR1* expression ➢ Directly targets *ESR1*	[126, 127]
miR-93	Suppresses *ESR1* expression	[11]
miR-103	Suppresses *ESR1* expression	[7]
miR-107	Suppresses *ESR1* expression	[7]
miR-122	Suppresses *ESR1* expression	[7]
miR-129	Suppresses *ESR1* expression	[7]
miR-130a/b	Suppresses *ESR1* expression	[7, 11]
miR-145	Suppresses *ESR1* expression, but does not affect *ESR1* or ERα stability ➢ Directly targets *ESR1*	[128]
miR-146b	Suppresses *ESR1* expression	[7]
miR-181a-d	Suppresses *ESR1* expression	[11]
miR-192	Suppresses *ESR1* expression ➢ Directly targets *ESR1*	[129]
miR-193b	Suppresses *ESR1* expression ➢ Directly targets *NCOA3*	[11, 125]
miR-206	Suppresses *ESR1* expression and ER target genes ➢ Directly targets *ESR1* ➢ Directly targets *NCOA1* and *NCOA3* ➢ Directly targets *GATA3* ➢ Directly targets *MET*	[1, 7, 11–14]
miR-219	Suppresses *ESR1* expression	[11]
miR-221	Suppresses *ESR1* expression ➢ Directly targets *ESR1*	[8, 11]
miR-222	Suppresses *ESR1* expression ➢ Directly targets *ESR1*	[7, 8, 11]
miR-301a/b	Suppresses *ESR1* expression	[11]
miR-302a-e	Suppresses *ESR1* expression	[11]
miR-335	Suppresses *ESR1* expression ➢ Directly targets *ESR1*	[130]
miR-372	Suppresses *ESR1* expression	[11]
miR-373	Suppresses *ESR1* expression	[11]
miR-517a/c	Suppresses *ESR1* expression	[11]
miR-520a-e	Suppresses *ESR1* expression	[11]
miR-548p	Suppresses *ESR1* expression	[7]
miR-583	Suppresses *ESR1* expression	[7]
miR-590	Suppresses *ESR1* expression	[7]
miR-643	Suppresses *ESR1* expression	[7]
miR-874	Suppresses *ESR1* expression	[7]
miR-885	Suppresses *ESR1* expression	[7]
miR-934	Suppresses *ESR1* expression	[7]
miR-1231	Suppresses *ESR1* expression	[7]

### miR-22

miR-22 is a classical example of the negative correlation between *ESR1*-targeting miRNA expression and ER status in breast cancer. As such, miR-22 is downregulated in ER^+^ breast cancer cell lines, as well as clinical samples [7]. Studies have also consistently reported that miR-22 can post-transcriptionally inhibit ERα expression/function by directly binding to the 3’ UTR of *ESR1* [7–9]. Indeed, the overexpression of miR-22 blocks ERα-dependent cell proliferation and ER-mediated transcriptional activity in vitro [9]. miR-22 was additionally found to indirectly inhibit ERα activity through the direct suppression of the ERα transcriptional coactivator, Sp1 [10]. The induction of cellular senescence by miR-22 overexpression also appeared to correlate with a less invasive cellular phenotype in human breast and cervical cancer cell lines [10]. These in vitro data were consistent with results demonstrating that miR-22 overexpression also promotes senescence and reduces the proliferative index of mammary tumor cells in a murine fat pad orthotopic mammary tumor model. In turn, these miR-22-induced cellular responses suppressed overall mammary tumor growth and reduced the appearance of metastatic tumors in vivo [10]. Together, the direct and indirect mechanisms of *ESR1*/ERα inhibition utilized by miR-22 suppress the oncogenic phenotypes associated with downstream ERα signaling activity, which makes miR-22 a promising candidate miRNA for therapeutic strategies to treat ER^+^ breast cancer.

### miR-206

miR-206 expression is observably lower in ER^+^ breast cancer versus ER^−^ breast cancer [1, 2]. Likewise, miR-206 is reported to directly target two binding sites in the 3’ UTR of *ESR1* in vitro [11–14]. ERα coactivators, *NCOA1*/SRC1 and *NCOA3*/SRC3, are also putative targets of miR-206 [14]. As well, miR-206 targets *GATA3*, a key marker for luminal breast cancer that can activate or be activated by ERα [14, 15]. Indeed, ectopic overexpression of miR-206 significantly represses the oncogenic phenotype associated with ER-mediated cell proliferation and survival, and induces G0/G1 cell cycle arrest in vitro [1, 11, 12, 14]. As a potential mechanism of miR-206-induced G0/G1 cell cycle arrest, it has been recently reported that miR-206 directly targets WBP2, which regulates multiple cell cycle proteins involved in G1 to S phase transition, such as p21, CDK4, and Cyclin D1 [16]. In addition to the suppression of ERα-mediated responses and cell cycle regulation, miR-206 also directly targets the 3’ UTR of *MET*, which is associated with aggressive breast cancer phenotypes [12, 17]. Together, these reports further validate the miR-206-induced effects on ERα expression/activation and ER-mediated breast carcinogenesis [14]. The cell cycle and growth-promoting targets of miR-206 also highlight the potential application of miR-206 expression restoration as a potential therapeutic strategy for ER^+^ breast cancer, particularly the luminal B subtype that is typically characterized by a high proliferative index.

### miR-221/222

Various studies have demonstrated that miR-221/222 target oncogenes, as well as tumor suppressor genes. Of particular importance in ER^+^ breast cancer, miR-221/222 targets *ESR1* [8, 11]. The negative regulation of *ESR1*/ERα has significant clinical implications related to acquired resistance to endocrine therapies, as discussed later in this review. On the other hand, miR-221/222 can indirectly promote ERα/estrogen signaling through direct inhibition of *BECN1*/Beclin1, which is a key regulator of autophagy. Beclin1 can induce autophagy and suppress the tumorigenic properties of MCF-7 breast cancer xenografts in vivo [18]. Although cytokine *mda-7*/IL-24-induced suppression of miR-221 has been found to result in Beclin1-associated autophagic cell death [19], Beclin1 acts as a negative regulator of ERα/estrogen signaling, which may interfere with the efficacy of anti-estrogen therapies [20].

In addition to the regulation of estrogen/ERα signaling, miR-221/222 have a number of tumor suppressor targets, which suggest the oncogenic functional mechanisms of miR-221/222. As a potential mechanism of miR-221/222-associated tumorigenesis, key cell cycle inhibitors have been identified as targets of miR-221/222, which contributes to oncogenic cell cycle progression. In particular, miR-221/222 are negative post-transcriptional regulators of *CDKN1B*/p27^*Kip1*^, a cell cycle inhibitor [21, 22]. As such, miR-221/222 overexpression suppresses p27^*Kip1*^ expression and correspondingly promotes G1/S transition in MCF-7 and MDA-MB-231 breast cancer cell lines [23, 24]*.* In vitro studies using breast and other cancer models have further corroborated the oncogenic role of miR-221/222 by demonstrating TIMP3, PTEN, and PUMA, as putative targets of miR-221/222 [25–29]. The inhibition of miR-221/222 targets induces effects on multiple signaling pathways, which results in a range of cellular responses involved in cell proliferation, apoptosis, and invasion/migration. TIMP3 exhibits anti-cancer actions via the inhibition of metalloproteinases, including several ADAMs, which has been shown to block cell migration and invasion associated with cancer cell metastasis. Moreover, PTEN is an inhibitor of the PI3K/Akt pathway and has been well-established as a tumor suppressor. In MCF-7 ER^+^ breast cancer cells, PTEN suppression via miR-221/222 overexpression was associated with PI3K/Akt-mediated promotion of cell proliferation, migration/invasion, and stemness/self-renewal [28]. Furthermore, PUMA is a pro-apoptotic protein that is regulated by p53. Zhang et al. (2010) found that miR-221/222 knockdown induced PUMA-dependent apoptosis in MCF-7 breast cancer and A549 lung cancer cells [29]. The pro-apoptotic function PUMA was further demonstrated by the concurrent induction of Bax and suppression of Bcl2.

In context with miR-221/222 expression and breast cancer in patients, recent reports indicate that miR-221/222 expression does not significantly correlate with overall and disease-free survival in breast cancer patients not grouped by subtype [30, 31]. However, according to a study by Han et al. (2017), high expression levels of miR-222 in patients with ER^+^ breast cancer were significantly (*P* = 0.021) associated with decreased disease-free survival as compared to ER^+^ breast cancer patients exhibiting low levels of miR-222 [31]. In contrast, the effect of miR-222 expression was not correlated with significant differences in disease-free survival of ER^−^ breast cancer patients. Thus, these reports provide supportive evidence that miR-221/222 are key regulators of ER^+^ breast carcinogenesis.

### miRNAs Regulated by Estrogen/ERα

Estradiol/estrogen signaling has been shown to regulate hundreds of genes through direct ERα binding site interaction, transcription factor activation, and miRNA regulation. miRNA microarray analyses have demonstrated that estradiol can upregulate the expression of numerous individual miRNAs and miRNA families, including the let-7, miR-17-92, and miR-200 family members (Table 3) [32]. In this section, several estradiol-regulated miRNAs will be highlighted and the functional implications of their regulation will be discussed in context with ER^+^ breast carcinogenesis.

**Table 3:** miRNAs regulated by estrogen/ERα activation

miRNA	Known mechanisms of regulation by estrogen/ERα	References
*miRNAs upregulated by estrogen/ERα*		
let-7a-g/i	Located in the intragenic region of an estradiol-regulated gene	[32, 57]
miR-7		[8, 39]
miR-9		[131]
miR-17	ERα-induced *MYC*/c-MYC upregulation; c-MYC interaction with the miR-17-92 promoter	[32, 39, 57, 59]
miR-18a		[57]
miR-19a/b		[8, 57]
miR-20a	ERα-induced *MYC*/c-MYC upregulation; c-MYC interaction with the miR-17-92 promoter	[8, 39, 57, 59]
miR-21	Contains ERα binding sites in regulatory region	[32, 39, 44, 57, 59]
miR-23a	Contains ERα binding sites in regulatory region	[32]
miR-24		[132]
miR-25		[39, 57]
miR-27b		[39, 67]
miR-29a		[39]
miR-30b/c	Located in the intragenic region of an estradiol-regulated gene	[32]
miR-32		[57]
miR-34c		[39]
miR-92a/b		[57, 59, 132]
miR-93	Requires estrogen metabolism-mediated oxidative stress	[57, 132, 133]
miR-98		[32, 57]
miR-99b		[132]
miR-101		[57]
miR-103		[32, 132]
miR-106a/b		[8, 57, 59]
miR-107		[32]
miR-124		[39]
miR-127		[39]
miR-129		[59]
miR-148a		[39]
miR-149		[39]
miR-181d		[39]
miR-191		[39, 132]
miR-196a	Contains ERα binding site in promoter region	[134]
miR-200a-c		[32, 132]
miR-203		[32]
miR-206		[39]
miR-210		[39]
miR-301a		[39]
miR-320a/c		[132]
miR-424		[32, 57]
miR-450		[57]
miR-489		[57]
miR-503		[135]
miR-542		[57]
miR-638		[132]
miR-1275		[132]
miR-1915		[132]
*miRNAs downregulated by estrogen/ERα*		
let-7a/c/f/g	Transcriptionally repressed by estradiol-induced ER	[40, 132]
miR-9	Located in the intragenic region of an estradiol-regulated gene	[32]
miR-15b		[132]
miR-21	Mediated by AF-1; Transcriptionally repressed by estradiol-induced ER; Contains ERα binding sites in promoter/regulatory region; Mediated by unliganded ERα	[40–44, 132]
miR-22		[57, 59]
miR-23b	Transcriptionally repressed by estradiol-induced ER	[40]
miR-26a/b		[40, 132]
miR-27a/b	Contains ERα binding sites in regulatory region; Transcriptionally repressed by estradiol-induced ER	[32, 40, 57]
miR-127		[59]
miR-143		[32]
miR-148a		[136]
miR-181a/b/d	Contains ERα binding sites in regulatory region; Transcriptionally repressed by estradiol-induced ER; Mediated by unliganded ERα	[40, 57, 137]
miR-198		[57]
miR-200c		[40]
miR-206		[13]
miR-221/222	Contains ERα binding sites in promoter region	[12]
miR-302b		[32]
miR-487b		[57]
miR-494		[57]
miR-500		[57]
miR-506		[32]
miR-519e		[8]
miR-524		[32]
miR-584		[57]
miR-663		[57]
miR-671		[57]
miR-1228		[8]
miR-1826		[132]

### miR-21

miR-21 is a well-studied oncomiR with anti-cancer targets, like PDCD4 [33], TIMP3 [34], and PTEN [35], and is overexpressed in human breast cancer tissues as compared to healthy breast tissues [34, 36]. Indeed, miR-21 expression levels increase with tumor grade and invasiveness of the cancer [37]. Thus, given the strong association between miR-21 expression and breast cancer, how miR-21 is regulated is an important question. A recent review by Petrović (2016) postulated that the oncogenic role of miR-21 in breast cancer may lie predominately in the regulation of cancer cell growth and invasion rather than the events that contribute to cancer initiation [38]. Moreover, miR-21 expression was reported to exhibit a 2-fold increase in ER^+^ breast cancer cells exposed to estradiol [32, 39]. However, in contrast to reports demonstrating that miR-21 is upregulated in response to estradiol, several studies have reported that estradiol suppresses miR-21 expression in vitro [40–43]. In context with the conflicting effects of estradiol on miR-21 expression, ERα binding sites found in the miR-21 regulatory region are key to determining the upregulation or downregulation of miR-21. In particular, Bhat-Nakshatri et al. (2009) found that unliganded ERα suppresses miR-21 expression, whereas ligand-bound/estradiol-activated ERα promotes miR-21 upregulation [32]. Additionally, overexpression of a truncated form of ERα (ERα46) may trigger estradiol-induced miR-21 upregulation [44]. ERα activator, AF-1, was also found to be an integral factor in estradiol-induced miR-21 suppression. Although in vivo data are still missing to fully understand and confirm the effects of estradiol on miR-21 expression, these contrasting reports indicate a complex regulatory mechanism between estradiol and miR-21 that may be differentially regulated at different breast cancer stages.

In regards to the potential of miR-21 as a target for breast cancer inhibition, studies using cell models of ER^+^ breast cancer have consistently reported that miR-21 inhibition suppresses ER^+^ breast carcinogenesis [45, 46]. Interestingly, several reports have indicated that non-pharmaceutical interventions can modulate miR-21 expression. For instance, exercise training can reduce the expression of miR-21, which was postulated to contribute to the decreased mammary tumorigenesis observed in in vivo ER^+^ breast cancer models [47, 48]. These studies found that exercise downregulated miR-21 and rescued the expression of miR-21 targets, including PDCD4. Moreover, Khori et al. (2015) found that exercise training was equally effective as tamoxifen in reducing estradiol levels and ERα expression, and both tamoxifen and exercise training significantly reduced tumor burden in a BALB/c mouse model of ER^+^ breast cancer [47]. Since PDCD4 downregulation is a major marker of poor prognosis and endocrine therapy resistance in ER^+^ breast cancer patients [49], the pharmacological, as well as non-pharmacological, suppression of miR-21 represents a promising treatment strategy for ER^+^ breast cancer patients; however, questions remain to be addressed regarding the regulatory mechanisms that control miR-21 expression during breast carcinogenesis, as well as the mechanism by which exercise training alters miR-21 expression. Uncovering these key mechanisms would significantly advance our understanding of how physiological responses can regulate miRNAs that are associated with oncogenic processes.

### miR-7

miR-7 expression is elevated in ER^+^ breast cancer cell lines and is associated with tumor aggressiveness, as indicated by the positive correlation between miR-7 expression and increased tumor size, grade, and metastasis in ER^+^ breast cancer patients [8, 50, 51]. In contrast, miR-7 expression is inversely correlated with the invasiveness of breast cancer cell lines in vitro [52]. Moreover, miR-7 is reported to influence the G2/M cell cycle checkpoint and DNA repair via inhibition of key regulators, such as *WEE1*, *GADD45A*, *TP53*, and *ATM* [50], which contributes to the reduced proliferative phenotype observed breast cancer cells overexpressing miR-7 [53]. As such, impaired cell cycle and DNA repair associated with miR-7 expression were found to be contributing factors for an accumulation of chromosomal instability. Several groups further explored the mechanisms that regulate miR-7 in ER^+^ versus ER^−^ breast cancer cell lines and determined that miR-7 is upregulated via estrogen-mediated ERα activation in vitro [8, 39]. As a potential regulatory mechanism that promotes ER dependency for cell proliferation and survival in ER^+^ breast cancer cells, receptor tyrosine kinases (RTKs), such as EGFR and IGF1R, and their associated ligands, including IRS2, are putative targets of miR-7 [8, 54–56]. However, Cui et al. (2017) recently found that miR-7 overexpression downregulated EGFR and IGF1R protein expression in ER^−^ MDA-MB-231 breast cancer cells, but not ER^+^ MCF-7 breast cancer cells [52]. Although estradiol mediates miR-7 upregulation to confer ER-dependent cell growth, miR-7 downregulation may contribute to endocrine therapy resistance due to the lack of RTK inhibition, which consequently activates compensatory oncogenic signaling pathways. Alternatively, in ER^−^ breast cancers, miR-7 overexpression has been considered as a potential therapeutic strategy to exploit miR-7-mediated suppression of EGFR and IGF1R, which are often overexpressed in triple-negative breast cancer (TNBC). Indeed, miR-7 overexpression can reduce the metastatic potential of TNBC cells in vitro [52]. Given the potential breast cancer subtype-specific effects of miR-7 expression, understanding the role of ER-associated regulation of miR-7 and its downstream targets will underscore the clinical applications of targeting miR-7 as novel therapeutic strategies for ER^+^ breast cancer patients, as well as TNBC patients.

### miR-17 and miR-17-92 Cluster

miR-17 is a member of the miR-17-92 cluster, which includes the miR-17, miR-19, and miR-92 families. The miR-17 family includes miR-17, miR-18a, and miR-20a; the miR-19 family includes miR-19a and miR-19b; and miR-92 belongs to its own family. Based on several studies, estradiol appears to be a major stimulator of many miRNAs in the miR-17-92 cluster. Specifically, miR-17 and miR-20a have been shown to be upregulated by 1.7–3.36 fold in estradiol-treated ER^+^ breast cancer cell lines via ERα-induced *MYC*/c-MYC upregulation [32, 39, 57, 58]. Consistent with these in vitro microarray data, Kovalchuk et al. (2007) reported that estradiol exposure upregulated miR-17, miR-20a, and miR-92 in a rat model of estradiol-induced breast carcinogenesis, which was accompanied by oncogenic changes in mammary gland morphology, including lobular hyperplasia with increased alveoli and duct numbers [59]. As well, c-MYC was also found to be significantly upregulated in estradiol-exposed rats, further supporting estradiol/ERα-induced c-MYC upregulation as a major regulatory mechanism of the miR-17-92 cluster. Estradiol-induced modulation of gene and histone methylation may also contribute to these associated changes in miRNA expression. Nevertheless, additional studies are necessary as there is a major gap in our understanding of the mechanisms that mediate estradiol/ERα-associated regulation of miRNAs.

miR-17 is often characterized as an oncomiR given its inhibition of putative targets involved in tumor suppression, including p21 [58, 60]. However, several genes that promote cell growth and survival, such as *CCND1*, *STAT3*, and *E2F1*, are also suppressed by miR-17, as well as miR-20a [58, 61–63]. Thus, miR-17 may have oncogenic or tumor suppressor functions that are dependent on specific cellular conditions that determine the need for cell survival. To note, AIB1 (also known as NCOA3 or SRC3) is a putative target of miR-17 that is implicated in breast carcinogenesis and endocrine therapy resistance, indicating that miR-17 may also function as a regulator of ERα signaling [64–66]. As well, it has recently been reported that induced overexpression of miR-17 can sensitize cells to tamoxifen-mediated apoptosis in vitro [63]. Given the evidence that miR-17 can exhibit functions as both an oncomiR and a tumor suppressor, additional studies are warranted to elucidate the microenvironmental/cellular context that may influence the cancer-promoting or anti-cancer properties of miR-17 and its related family of miRNAs.

### miR-27a/b

Reports examining the effects of estradiol on miR-27a/b expression have produced conflicting data. For instance, microarray data have indicated that estradiol can elicit a 0.6 to 2.65 fold change in miR-27a/b expression in MCF-7 cells [32, 39, 40, 67]. Despite conflicting in vitro reports, Tang et al. (2012) confirmed that high miR-27a expression and low ZBTB10 expression, a putative target of miR-27a, were associated with poor patient survival [68]. In context with the regulatory role of miR-27a/b in ER^+^ breast cancer, *ZBTB10* is a major inhibitor of Sps, including Sp1 [69, 70]. Sp1 interactions with ERα are integral for ERα transcriptional activity; therefore, the indirect regulation of Sp1 via miR-27a may direct the activation or inhibition of ERα activity. miR-27b is also shown to target *CYP1B1*, a gene that encodes for an enzyme that is important for the hydroxylation of estradiol, which can produce free radicals and lead to DNA damage [71]. Since miR-27b expression is reported at higher levels in normal breast tissues as compared to breast cancer tissues [71], the suppression of miR-27b by estradiol [32] may be a cancer-promoting mechanism that results in the increased expression of CYP1B1 and subsequent DNA damage. Moreover, miR-27b levels are also reduced in tamoxifen-resistant breast cancer cell lines as well as patient samples, which are associated with the increased expression of miR-27b targets *NR5A2/LRH1* and *CREB1* [67]. The upregulation of *NR5A2* has a particularly important oncogenic function as it directly binds to the ERα promoter to stimulate transcription [72] and promote MYC expression [73], which has previously been reported to upregulate the expression of other oncomiRs (i.e. miR-17 and miR-20a) [58]. Thus, further studies are needed to dissect the factors that regulate the balance between the physiological and oncogenic functions of miR-27a/b.

### Feedback Mechanisms that Regulate Estrogen/ER Signaling

**Fig. 1:**
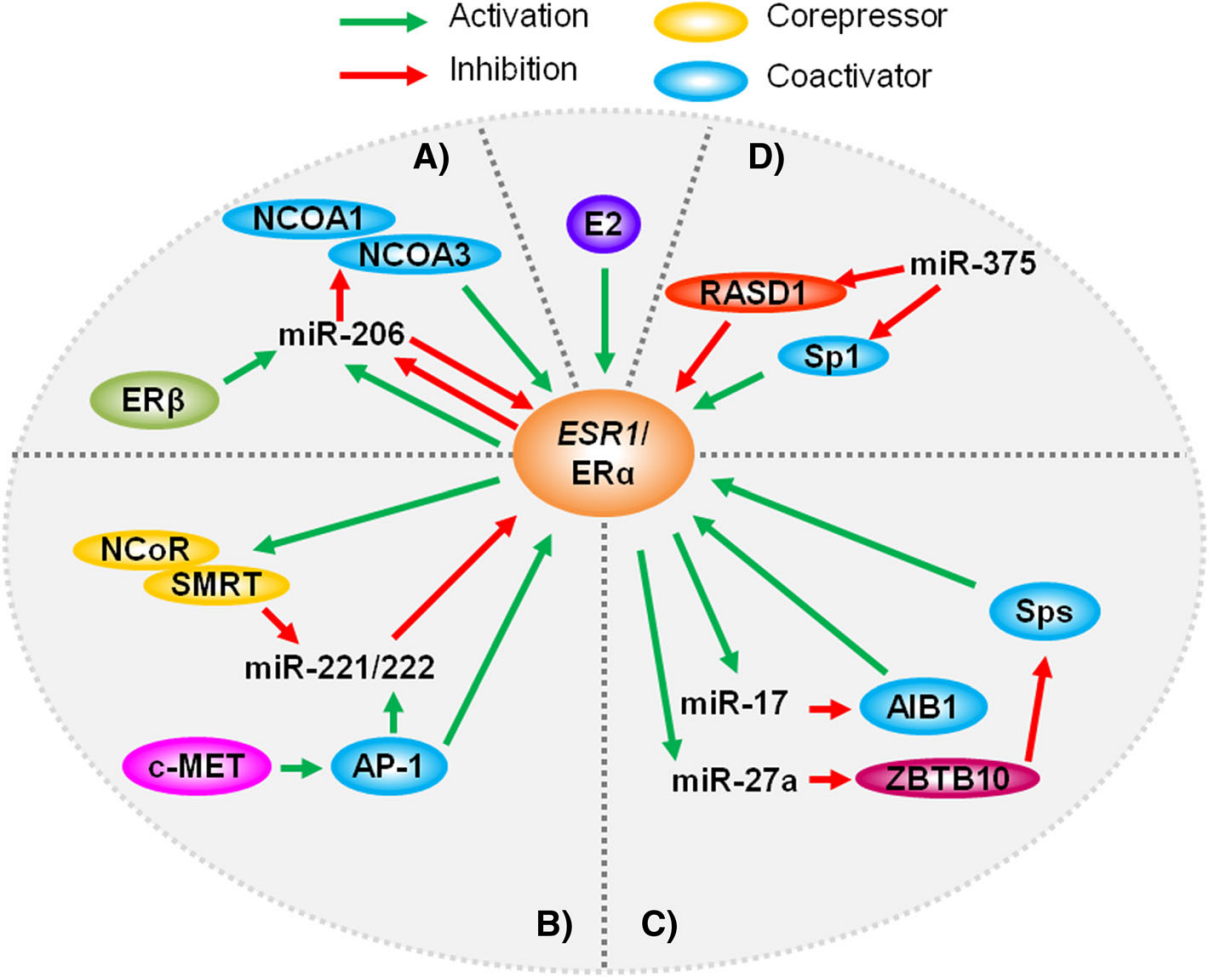
Feedback mechanisms that regulate estrogen/ERα signaling. miR-206 (**a**), miR-221/222 (**b**), miR-17 and miR-27a (**c**), and miR-375 (**d**) are involved in regulatory mechanisms that can contribute to the activation or inhibition of estrogen/ERα signaling

Although it has been well-established that miR-206 directly targets the 3’ UTR of *ESR1*/ERα in ER^+^ breast cancer cells [1, 11, 12], Adams et al. (2007) identified a feedback mechanism where estradiol and other ERα agonists can significantly inhibit miR-206 expression, while ERβ agonists promoted miR-206 expression (Fig. 1a) [13]. In contrast, microarray data reported by Masuda et al. (2012) found that estradiol markedly upregulated miR-206 by 3.35-fold in MCF-7 cells, which would consequently inhibit ERα expression and activity [39]. Lee et al. (2013) further suggested a functional role of ERα-mediated miR-206 regulation during physiological mammary gland development as miR-206 was found to be repressed downstream of ERα, indicating a potential role in ductal growth regulation [74]. Other estrogen/ER-associated miRNAs may also participate in regulatory loops to modulate estrogen/ER signaling. miR-221/222, which directly target *ESR1*, are other key examples as ERα knockdown in MCF-7 cells revealed that miR-221/222 can be regulated by ERα activity in vitro (Fig. 1b) [12]. Specifically, estrogen-mediated ERα activation recruited corepressors NCoR and SMRT to the transcription start site of miR-221/222. Garofalo et al. (2009) also proposed a mechanism of miR-221/222 upregulation in non-small cell lung cancer (NSCLC) and hepatocellular carcinoma (HCC) models involving c-MET/MET-mediated upregulation of the transcription factor AP-1 [25]. This mechanism may be potentially translated to miR-221/222 upregulation associated with ER^+^ breast cancer models; however, further in vitro and in vivo evidence is warranted.

As previously discussed, miR-17 and miR-27a/b may also be involved in estrogen/ERα pathway regulation. The expression of these miRNAs has been found to be upregulated by estradiol/ERα in numerous reports [32, 39, 58, 59]; however, the attenuation of their putative targets involved in ERα regulation, such as *AIB1* by miR-17 and *ZBTB10* by miR-27a, results in indirect ERα signaling inhibition and activation, respectively (Fig. 1c) [64, 67, 69]. Additionally, miR-375 is reportedly involved in a feedback mechanism that regulates ERα expression and signaling. Simonini et al. (2010) found that hypomethylation of the miR-375 promoter epigenetically stimulated miR-375 expression, which was positively correlated with ERα expression and activity [75]. The authors further identified *RASD1*, a negative regulator of ERα, as a direct target of miR-375 (Fig. 1d). In contrast, others have reported that miR-375 can directly target Sp1, an ERα coactivator, in HCC, colorectal, and cervical cancer models [76–78]. Although miR-375-mediated Sp1 regulation has not been verified in breast cancer models, this potential mechanism highlights a facet of miR-375-associated estrogen/ERα signaling regulation. Collectively, miRNA involvement in positive/negative feedback loops that regulate ERα activity can, at times, produce contradictory cellular responses, which further underscores the complexity of these regulatory networks that need to be unraveled to advance the potential development of miRNA-targeting therapeutics for ER^+^ breast cancer.

## miRNAs and Drug Resistance

In ER^+^ breast cancer, several therapeutic strategies are employed to selectively inhibit estrogen/ERα signaling, including ER antagonists that directly bind to ERα and enzyme inhibitors that block estrogen synthesis. However, de novo and acquired resistance to conventional endocrine therapies can occur in more than 30% of patients [79, 80]. Due to general targeting of estrogen/ER signaling, cross-resistance to multiple endocrine therapies is also a major concern. Therefore, elucidating the mechanisms of therapeutic resistance is integral to preventing and overcoming this significant clinical challenge. Several mechanisms of endocrine therapy resistance have been identified, including decreased ER expression, ER-independent signal transduction, and even miRNA regulation. Notably, miRNAs are promising targets for the novel therapeutic strategies due to the broad range of mRNA targets that ultimately regulate many of the ER-independent mechanisms associated with endocrine therapy resistance. In this section, resistance to tamoxifen, fulvestrant, and aromatase inhibitors will be discussed in context with miRNAs that may confer the resistant phenotype and/or may be promising therapeutic targets to increase patient responsiveness to the first-line endocrine therapies.

### Tamoxifen Resistance

As discussed in the previous section, although miR-221/222 are shown to target and inhibit the translation of oncogenic proteins associated with ER^+^ breast cancer, several studies have linked miR-221/222 expression to tamoxifen resistance. Miller et al. (2008) determined that miR-221/222 expression was positively correlated with resistance to tamoxifen-mediated cell growth inhibition, apoptosis, and cell cycle inhibition in MCF-7 breast cancer cells [23]. In context with the 3’ UTR of CDKN1B mRNA as a known target of miR-221/222 [21, 22, 24], *CDKN1B* mRNA/p27^*Kip1*^ protein regulation appears to be an essential mediator of miR-221/222-induced tamoxifen resistance in MCF-7 cells. Indeed, ectopic overexpression of p27^*Kip1*^ rescues tamoxifen sensitivity in MCF-7 cells [23]. A report by Wei et al. (2014) suggested that exosomal secretion of miR-221/222 may be a mechanism that confers tamoxifen resistance [81]. Interestingly, secreted exosomes from tamoxifen-resistant MCF-7 cells contained miR-221/222 and could be transferred into tamoxifen-sensitive cells, which rendered the cells resistant to tamoxifen-induced cell growth inhibition and apoptosis. Consistent with miR-221/222 upregulation, ERα and p27^*Kip1*^ protein levels were also decreased in the MCF-7 cells exposed to exosomes from tamoxifen-resistant cells. As another potential mediator of tamoxifen resistance, miR-21 was found to be activated by 4-hydroxytamoxifen (4-OHT)- and fulvestrant-mediated blockage of estrogen/estradiol [41]. This in vitro study presented evidence of miR-21 expression as a resistance factor in ER^+^ breast cancer; nevertheless, additional studies are warranted to fully understand the oncogenic mechanisms of miR-21 and miR-221/222 associated with therapeutic resistance.

ERα expression is a critical factor that mediates tamoxifen resistance. As such, ERα suppression by miRNAs has been shown to block the efficacy of tamoxifen. In this regard, the expression levels of *ESR1*/ERα-targeting miRNAs, such as miR-221/222 [8], miR-18a [82], miR-19a/b [83], and miR-22 [9], may predict the clinical responsiveness of ER^+^ breast cancer patients to tamoxifen. Moreover, Beclin1-overexpressing MCF-7 cells exhibited estradiol-induced colocalization of Beclin1 and ERα, which contributed to decreased sensitivity to tamoxifen derivatives, raloxifene and 4-OHT [20].

### Tamoxifen Sensitization

In recent years, several strategies have been proposed to increase breast cancer cell sensitivity to tamoxifen. First, targeted inhibition of miRNAs associated with tamoxifen resistance has provided in vitro evidence of increased tamoxifen sensitivity in ER^+^ breast cancer cells. As such, miR-221/222 knockdown upregulated TIMP3 mRNA and protein expression, which corresponded with enhanced tamoxifen-induced inhibition of cell viability in MCF-7 ER^+^ breast cancer cells [26]. To note, miR-221/222 inhibition did not affect the responsiveness of MDA-MB-231 triple-negative breast cancer cells to tamoxifen, which is consistent with the link between miR-221/222 expression, estrogen/ERα signaling, and tamoxifen activity. Furthermore, miR-221/222 silencing in tamoxifen-resistant MCF-7 cells protected wild-type cells from exosomal miR-221/222-induced tamoxifen resistance [81].

The inhibition of known oncomiRs, such as miR-21, has also provided a promising strategy to suppress ER^+^ breast carcinogenesis and enhance the effects of tamoxifen and other anti-estrogen treatments. Recently, Yu et al. (2016) reported that ectopic suppression of miR-21 via transfection with a miR-21 inhibitor results in Beclin1-mediated autophagic cell death, as well as increased sensitivity to both tamoxifen and fulvestrant, as demonstrated by an increased induction of apoptosis in vitro [46]. Moreover, nanoparticles co-loaded with anti-miR-21 and 4-OHT have shown promising anti-proliferative effects on MCF-7 ER^+^ breast cancer cells in vitro [84]. As compared to the 4-OHT treatment alone, the anti-miR-21 + 4-OHT nanoparticles induced a significant decrease in cell proliferation, which was accompanied by cell cycle arrest in G2/M phase. Furthermore, miR-34a expression has been reported to suppress miR-21 via the post-transcriptional inhibition of CD24 and Src, which were found to induce miR-21 expression, in in vitro breast and colon cancer models [85]. Importantly, CD24 expression has been positively correlated with ERα and ErbB2 status in breast cancer [86], and Src is involved in the phosphorylation/activation and intracellular localization of ERα and subsequent DNA synthesis [87]. Together, these data provide supportive evidence for testing miR-34a mimics and/or miR-21 inhibitors to treat ER^+^ breast cancer and to overcome tamoxifen resistance.

Alternatively, the induced upregulation of miRNAs that are reportedly downregulated in tamoxifen-resistant cells has been shown to significantly improve ER^+^ breast cancer cell sensitivity to tamoxifen. For instance, miR-378 is downregulated in tamoxifen-resistant MCF-7 cells and is found at lower levels in human breast cancer tissues as compared to normal adjacent tissues [88]. Low miR-378 expression was further associated with decreased recurrence-free survival in ER^+^ breast cancer patients taking tamoxifen as an adjuvant therapy [88]. Importantly, key mechanistic data suggest that miR-378-mediated inhibition of GOLT1A, which is involved in the fusion of transport vesicles with the Golgi membrane, plays a critical role in tamoxifen resensitization in vitro, which is further corroborated by clinical data demonstrating increased relapse-free survival in breast cancer patients with low GOLT1A expression. miR-27b is also reportedly downregulated in tamoxifen-resistant ER^+^ breast cancer cells and patient tumor tissue samples [67, 89]. Zhu et al. (2016) showed that miR-27b-3p is downregulated in tamoxifen-resistant ER^+^ breast cancer cells [67]. Interestingly, studies have reported conflicting miR-27b regulation by estrogen/estradiol. As previously mentioned, miR-27b was identified as a miRNA that is repressed by estradiol by 0.6 fold in MCF-7 cells [32]. In contrast, a recent report found that estradiol upregulated miR-27b-3p expression, and tamoxifen dose- and time-dependently suppressed miR-27b-3p expression in MCF-7 and T47D cells [67]. Moreover, overexpression via miR-27b mimics significantly enhanced ER^+^ breast cancer cell sensitivity to tamoxifen-induced cell growth inhibition and apoptosis. miR-27b overexpression also rendered tamoxifen-resistant breast cancer cells sensitive to tamoxifen in both in vitro cell line and in vivo xenograft tumor models [67]. The upregulation of miR-27b sensitization to tamoxifen in ER^+^ breast cancer cells was further associated with the suppression of direct targets, NR5A2/LRH1 and CREB1, which are implicated in tamoxifen resistance via the induction of ERα and aromatase, respectively [72, 90]. The negative correlation between miR-27b and NR5A2 and CREB1 expression has further been corroborated in human breast cancer tissues from tamoxifen-untreated and tamoxifen-resistant patients, suggesting that miR-27b may be a predictive marker for tamoxifen responsiveness, as well as a potential therapeutic target to overcome tamoxifen resistance [67].

Modulated miR-342 expression is also correlated with tamoxifen resistance/sensitivity in ER^+^ breast cancer. Numerous studies have consistently reported that miR-342 is downregulated in tamoxifen/4-OHT-resistant breast cancer cell lines and clinical samples [23, 91, 92]. In particular, miR-342 expression was reduced by 55% in 4-OHT-resistant MCF-7 cells as compared to parental cells [23]. Likewise, miR-342 downregulation is associated with tamoxifen treatment failure and decreased overall survival in ER^+^ breast cancer patients [30, 91]. In preclinical models of tamoxifen resistance, restoring the expression of miR-342 markedly sensitized ER^+^ breast cancer cells to tamoxifen-mediated cell growth inhibition and apoptosis [91]. Interestingly, He et al. (2013) reported that miR-342 expression is positively correlated with ERα expression in clinical breast cancer samples and breast cancer cell lines, which is proposed as a potential mechanism of enhanced tamoxifen sensitivity in breast cancer cell lines with ectopic miR-342 overexpression [92]. Together, these reports indicate that miR-342 is not only a potential biomarker for ER^+^ breast cancer patient survival and tamoxifen efficacy, but also may serve as a therapeutic target to enhance patient responses to tamoxifen.

The overexpression of EGFR, ErbB2, and other RTKs is linked to tamoxifen resistance as compensatory oncogenic signaling pathways [93, 94]. Therefore, targeted inhibition of these pathways has been presented as a potential strategy to overcome tamoxifen resistance. For example, miR-26a/b are reported to target the 3’ UTR of *ERBB2* and suppress ErbB2 protein expression in tamoxifen-resistant ER^+^ breast cancer cells. In particular, miR-26 is suggested to be suppressed by estrogen/estradiol [95] and is found to be downregulated in tamoxifen-resistant breast cancer cells. However, high miR-26a expression is associated with decreased expression of known cell cycle regulatory targets, including Cyclin E1, EZH2, and Cdk1, and positive clinical outcomes in patients with metastatic breast cancer taking tamoxifen [96]. Overexpression of the tumor suppressor miR-34a has also been reported to suppress ErbB2 expression and breast cancer cell growth and invasion [97]. Given the oncogenic function of ErbB2 in acquired tamoxifen resistance, miR-34a expression may be a prognostic target to predict tamoxifen responsiveness in ER^+^ breast cancer patients. In addition, the knockdown of Beclin1, a target of miR-221 [19], has been reported to suppress ErbB2 expression and restore tamoxifen sensitivity in resistant MCF-7 cells in vitro [98]. Consistently, clinical data from ER^+^ breast cancer patients taking tamoxifen have demonstrated that high Beclin1 expression is correlated with decreased patient survival [98]. Based on reports highlighting the oncogenic role of Beclin1 in ER^+^ breast cancer, miR-221-induced inhibition of Beclin1 would theoretically inhibit ER^+^ breast carcinogenesis and promote tamoxifen sensitivity in ER^+^ breast cancers. Nevertheless, the anti-cancer mechanism involving Beclin1 knockdown, in context with reports indicating the cancer-promoting activities of miR-221/222, further adds to the complex role of miR-221/222 in ER^+^ breast cancer promotion and inhibition.

### Fulvestrant Resistance

Due to the different anti-cancer mechanisms of tamoxifen and fulvestrant (also known as ICI-182,780 or Faslodex), Zhou et al. (2018) recently reported that miRNA profiles generally differ between fulvestrant- and tamoxifen-resistant MCF-7 breast cancer cells [99]. Nevertheless, there are some miRNAs that are associated with resistance to both therapeutic agents because of the broad range of mRNA targets. As such, miR-21 overexpression has also been found to confer tamoxifen and fulvestrant resistance through the inhibition of its direct targets, including PTEN [46]. Interestingly, treatment with fulvestrant can upregulate miR-21 expression [49], which indicates that miR-21 may have a critical role in the development of fulvestrant resistance.

Other examples of miRNAs that are involved in both tamoxifen and fulvestrant resistance are miR-221/222. miR-221/222 are upregulated in both tamoxifen- and fulvestrant-resistant MCF-7 cells, but to a greater extent in the fulvestrant-resistant cell line, and confer the resistant phenotype [100]. Similar to the report indicating that exosomes can transfer miR-221/222 to promote tamoxifen resistance in nearby cells, microvesicles containing miR-221 produced from cancer-associated fibroblasts can transfer fulvestrant resistance to non-tumor cells in an IL6-dependent manner [101]. Fulvestrant, but not tamoxifen, also significantly upregulates miR-221/222 expression in parental MCF-7 cells, which may be an indicator of the development of fulvestrant-resistance. As well, miR-221/222 upregulation promotes fulvestrant resistance via multiple pathways, including the targeted inhibition of ERα and p27^*Kip1*^. The inhibition of ERα upon miR-221/222 overexpression consequently induces ERα-independent cell growth, which is a mechanism that confers fulvestrant resistance. RTK and Wnt/β-catenin pathways have been implicated in ERα-independent cell growth in fulvestrant-resistant cells [100, 102]. Particularly, the expression of critical genes involved in the Wnt/β-catenin pathway, including *CTNNB1*, *LRP6*, and *WNT11*, was previously found to be upregulated in fulvestrant-resistant MCF-7 cells, which coincided with hypomethylation of their gene promoters [102]. Moreover, β-catenin overexpression was found to confer fulvestrant resistance, but to a lesser extent than miR-221/222 overexpression, suggesting that miR-221/222 regulation of other targets is necessary to induce fulvestrant resistance. Together, these reports indicate that perhaps miR-221/222 may functionally regulate Wnt/β-catenin signaling through an epigenetic mechanism that modulates promoter methylation.

More recently, the activation of other oncogenic signaling pathways has been connected to miR-221/222 expression and acquired fulvestrant resistance, including the upregulation of key markers in the TGFβ, Notch, Jak-STAT, MAPK, and p53 signaling pathways [100]. Furthermore, miR-221/222 overexpression promoted TGFβ-induced cell survival in parental MCF-7 cells, whereas miR-221/222 knockdown enhanced the tumor suppressor activities of TGFβ with significantly decreased cell survival in fulvestrant-resistant MCF-7 cells. Thus, Rao et al. (2011) postulated that miR-221/222 may have an additional regulatory role that preferably stimulates the oncogenic function of TGFβ to promote fulvestrant-resistant breast cancer cell growth [100].

### Fulvestrant Sensitization

Integrative computational analyses of miRNA-gene expression in endocrine therapy-resistant breast cancer cell lines have revealed a miR-222-*CDKN1B*/p27^*Kip1*^ expression network that is conserved in both tamoxifen-resistant and fulvestrant-resistant MCF-7 cells [103]. Similar strategies to enhance ER^+^ breast cancer cell sensitivities to tamoxifen and fulvestrant are being tested. The knockdown of oncogenic miR-221/222 in fulvestrant-resistant MCF-7 cells resulted in the concurrent inhibition of cell growth and colony formation, as well as the upregulation of p27^*Kip1*^ upon fulvestrant treatment [100]. In addition to the well-studied regulatory function of miR-221/222 on p27^*Kip1*^ and ERα expression, mRNA expression profiling revealed that 428 gene probes were upregulated and 224 gene probes were downregulated in response to miR-221/222 knockdown in fulvestrant-resistant MCF-7 cells. These data highlight the widespread and complex functional role of miR-221/222 in fulvestrant resistance, and uncover novel pathways or key mediators, such as Wnt/β-catenin, TGFβ, EGFR, and ErbB2, that may be valuable targets for future therapeutic development or combined treatment strategies. Importantly, the targeted inhibition of EGFR, ErbB2, and β-catenin in tamoxifen-resistant and fulvestrant-resistant cells has demonstrated sensitization to the growth inhibitory effects proportional to the degree of phosphorylation/activation of these targets in the parental and resistant cells [102]. Given the correlation between these oncogenic compensatory pathways that promote ERα-independent cell growth and tamoxifen/fulvestrant resistance, the targeted inhibition of miR-221/222 expression is a promising strategy to prevent the acquisition of fulvestrant resistance and even treat fulvestrant-resistant breast cancer patients.

The regulation of autophagy is emerging as another potential strategy to overcome fulvestrant resistance. It was recently reported that the combined inhibition of EGFR/ErbB2 (via lapatinib) and c-ABL (via imatinib) in fulvestrant-resistant breast cancer cells significantly inhibited cell growth and autophagy [104]. These anti-cancer effects were found to be associated with the upregulation of miR-375, which has previously been linked to PDK1/Akt/mTOR-independent inhibition of autophagy, as indicated by decreased autophagosome formation, *ATG7* gene expression, and LC3-II protein expression, in HCC in vitro and in vivo [76]. In contrast to the anti-cancer effects resulting from miRNA-induced suppression of autophagy in fulvestrant-resistant cells, a report using parental MCF-7 cells found that miR-21 knockdown increased autophagic cell death via activation of its direct target PTEN and subsequent inhibition of the PI3K/Akt/mTOR pathway to enhance sensitivity to tamoxifen and fulvestrant [46]. To note, the effects of miR-21 knockdown on autophagy in resistant breast cancer cells has not been investigated. Moreover, increased autophagy has been reported in cells treated with 4-OHT and fulvestrant, which may be a potential mechanism that leads to the acquisition of therapeutic resistance [105]. miR-214 overexpression was shown to suppress 4-OHT- and fulvestrant-induced autophagy in MCF-7 cells through the inhibition of its direct target UCP2. Importantly, miR-214-mediated suppression of UCP2 led to the inhibition of the PI3K/Akt/mTOR pathway [105]. Based on these findings, the regulation of autophagy may have different consequences in context with the therapeutic sensitivity/resistance of the cells, which warrants further investigation. Advancing our understanding of the role of autophagy in fulvestrant/tamoxifen resistance will support the development of novel strategies that modulate autophagy to prevent or overcome endocrine therapy resistance.

### miRNA-Regulated Pathways Associated with Aromatase Inhibitor (AI) Resistance

AIs, such as letrozole and anastrozole, are typically prescribed to postmenopausal ER^+^ breast cancer patients. However, the prolonged estrogen deprivation in these patients can lead to acquired AI resistance, which is a critical clinical challenge. Emerging studies have determined that, like tamoxifen and fulvestrant resistance, AI resistance can also be mediated by miRNAs in ER^+^ breast cancer. Although the number of studies examining the connection between AI resistance and miRNA regulation in ER^+^ breast cancer is generally limited, several specific pathways appear to be the main targets associated with miRNA-mediated regulation of AI resistance.

According to a miRNA microarray using letrozole- and anastrozole-resistant MCF-7 cells, Vilquin et al. (2015) reported that both miRNA signatures in the resistant cells were associated with the regulation of the various signaling pathways, including MAPK signaling, focal adhesion, insulin signaling, ErbB signaling, and mTOR signaling pathways that converged on Akt regulation [106]. The microarray analyses determined that miR-125b, miR-205, and miR-30a were all significantly upregulated, while miR-424 was significantly downregulated, in both AI-resistant cell lines as compared to the sensitive cells. Interestingly, miR-125b and miR-205 overexpression and miR-424 suppression were able to confer resistance to both letrozole and anastrozole, as well as promote mammosphere/stem cell self-renewal, which may contribute to the aggressive nature of the resistant cells [106]. The activation of the PI3K/Akt/mTOR pathway appeared to be a critical mechanism of miR-125b/miR-205/miR-424-mediated AI resistance. In contrast, the targeted suppression of miR-125b/miR-205 and overexpression of miR-424 may rescue the sensitivity of the cells to AIs. Clinical data demonstrating that miR-125b overexpression is correlated with decreased relapse-free survival in primary ER^+^ breast cancer samples further support the role of miRNAs, particularly miR-125b, miR-205, and miR-424, in AI-resistance [106]. Compensatory activation of ErbB2 and downstream MAPK and Akt signaling in AI-resistant ER^+^ breast cancer cells resulted in the upregulation of miR-21 and downregulation of its target PDCD4 [49]. In clinical cohorts of ER^+^ breast cancer patients, PDCD4 expression is strongly linked to patient outcomes, as demonstrated by patients with low PDCD4 expression levels having poor disease-free survival and increased tumor grade. As PDCD4 is a direct target of miR-21, targeted inhibition of miR-21 significantly reduced cell proliferation in AI/letrozole-resistant MCF-7 cells in vitro [49], demonstrating that miR-21 may also be a potential target to treat AI resistance. Collectively, these preclinical and clinical data provide promising evidence regarding the potential clinical application of the targeted regulation of these miRNAs to overcome AI resistance and underscore the functional contribution of PI3K/Akt/mTOR pathway activation in AI resistance.

The TGFβ signaling pathway is another major pathway that can be modulated by miRNAs to promote AI resistance. Particularly, TGFβRI is identified as a direct target of miR-128a, which is overexpressed in letrozole-resistant MCF-7 cells [107]. TGFβ induces a growth inhibitory effect in letrozole-sensitive cells, but does not significantly alter cell growth in the resistant cells, suggesting that the miR-128-mediated regulation of TGFβRI may contribute to the differential responses in the letrozole-sensitive and -resistant cell lines. In turn, suppression of miR-128a was found to inhibit TGFβRI in letrozole-resistant cells, which promoted the growth inhibitory effects of TGFβ. As the correlation between miR-128a expression and letrozole resistance has only been reported in in vitro cell models, further examination in additional preclinical models and verification in clinical datasets are needed to substantiate the clinical value of miR-128a as a potential therapeutic target or marker for AI resistance.

Moreover, AI-resistant MCF7-LTED (long-term estrogen deprivation – an in vitro model of AI resistance) cells characteristically utilize glycolytic metabolism, but can switch to oxidative phosphorylation when the glycolytic pathway is impaired. The ability of AI-resistant cells to undergo this metabolic pathway reprogramming has been linked to estradiol-mediated upregulation of miR-155 [108]. Therefore, the expression of miR-155, which is reported to target hexokinase 2 (HK2) through the direct inhibition of miR-143 in head and neck squamous cell carcinoma models, is implicated in clinical AI resistance [108, 109]. To note, HK2 is a key primer for glycolysis. Indeed, targeting the miR-155/miR-143-HK2 axis may be a promising strategy to overcome letrozole resistance. As well, miR-155 expression may serve as a prognostic biomarker to predict the clinical responsiveness to AIs in women with ER^+^ breast cancer.

## Mechanisms that Regulate miRNA Biogenesis in ER^+^ Breast Cancer

As discussed previously, estrogen/ERα signaling can modulate the expression of various oncogenic and tumor suppressor miRNAs. However, the specific regulatory mechanisms have not been fully uncovered. In particular, the regulation of miRNA biogenesis and its functional machinery is a key mechanism that controls miRNA expression. In this section, the mechanisms of regulation and consequences of deregulated miRNA biogenesis will be discussed in context with ER^+^ breast carcinogenesis.

The process of miRNA biogenesis and the components that form essential complexes, such as Drosha, DGCR8, Dicer1, and Ago2, have been extensively studied. Particularly, the oncogenic consequences of miRNA biogenesis deregulation have been well-documented [110–112]. Estrogen/ERα can modulate key machinery involved in miRNA biogenesis, which may have key functional consequences in ER^+^ breast cancer. For instance, Dicer, a cytoplasmic endonuclease required for cleavage of the hairpin loop from the pre-miRNA, can be induced by estradiol as Dicer is reported to associate with an ERα binding site, which promotes *Dicer* mRNA transcription [32]. Accordingly, Dicer expression is upregulated in ER^+^ breast cancer [4]. To note, *Dicer* expression can be modulated by miRNAs, including miR-221/222, miR-29a, and miR-200c [8]. Conversely, Ago1/2 were found to be downregulated in ER^+^ breast cancers versus ER^−^ breast cancers despite upregulation of other components of the RISC (i.e. Dicer and TRBP) [4, 113]. Drosha expression was also significantly lower in ER^+^ breast cancer samples. As well, it has been proposed that activated ERα may interfere with the Drosha-DGCR8 microprocessor complex that is responsible for pri-miRNA cleavage [114]. Nevertheless, as a potential functional consequence of estradiol-mediated Dicer induction and the activation of other cell signaling pathways that stimulate miRNA biogenesis, the overall expression level of miRNAs is significantly increased in ER^+^ breast cancer cell lines and patient samples [4, 8]. Given the differential expression/regulation of miRNA processing machinery in ER^+^ breast cancer, it is critical to understand the factors that regulate these key components and their specific role in cancer promotion or suppression. Overall, defective miRNA biogenesis and machinery appears to be an important mechanism that ultimately contributes to ER-associated breast carcinogenesis. Thus, further exploration of these regulatory mechanisms is necessary to advance our understanding of how miRNA gene regulation can contribute to breast cancer, and may also reveal novel therapeutic strategies/targets for treating ER^+^ breast cancer.

## Role of miRNAs in ER-RTK Crosstalk Regulation in Breast Cancer

Studies unraveling the complex oncogenic signal transduction networks associated with breast cancer initiation and progression have revealed that crosstalk between key signaling pathways is an important factor contributing to breast carcinogenesis and resistance to therapeutic agents. In particular, crosstalk between the ER and RTK pathways has emerged as a critical mechanism of compensatory pathway activation, particularly in endocrine therapy-resistant ER^+^ breast cancers [115]. The RTK family includes a number of growth factor-mediated receptors, such as IGF1R, EGFR, and ErbB2/Her2, that are reported to promote several hallmarks of breast cancer. Arpino et al. (2005) found that high ErbB2/Her2 and EGFR expression was independently associated with a significant decrease in disease-free survival in ER^+^ breast cancer patients taking tamoxifen [116]. Importantly, studies have found that several miRNAs can regulate targets involved in both the ER and RTK pathways, thus repressing ER-RTK crosstalk. Of particular importance, miRNAs involved in the regulation of ER-RTK crosstalk may serve as leading candidates for the development of novel miRNA-based breast cancer therapies targeting multiple oncogenic pathways.

## Summary and Future Perspectives

Our review of miRNA-associated hormonal signaling in ER^+^ breast cancer has revealed a complex signaling network that can regulate ER^+^ breast cancer progression and response to anti-estrogen therapies. Although substantial in vitro data in conjunction with human miRNA/mRNA expression profiles have uncovered key mechanisms of miRNA regulation, functional consequences, and potential therapeutic targets, further in vivo evidence from animal models of ER^+^ breast cancer are necessary to support the clinical application and development of miRNA-targeted therapeutics. These advances will ultimately help to identify prospective miRNA candidates for nucleic acid-based therapeutics, such as antagomiRs or miRNA sponges to suppress oncomiRs and miRNA mimics to promote the tumor suppressor miRNAs, which will further guide the clinical development and application of miRNA-based therapeutic strategies for the treatment of ER^+^ breast cancer. Advancing our mechanistic understanding of de novo and acquired resistance to endocrine therapies and miRNA regulation will also facilitate the identification of miRNA biomarkers and the development of miRNA inhibitors/activators to enhance the efficacy of current endocrine therapies and overcome therapeutic resistance in ER^+^ breast cancer patients.

## Abbreviations

4-OHT: 4-hydroxytamoxifen
AI: Aromatase inhibitor
ER^+^: Estrogen receptor-positive
ERα: Estrogen receptor alpha
HCC: Hepatocellular carcinoma
HK2: Hexokinase 2
LTED: Long-term estrogen deprivation
miRNA: microRNA
NSCLC: Non-small cell lung cancer
RTK: Receptor tyrosine kinase
SERD: Selective estrogen receptor downregulator
SERM: Selective estrogen receptor modulator
TNBC: Triple-negative breast cancer
